# Clinical and Economical Outcomes Associated with Parathyroidectomy: A 5-Year Population-Based Study in a Middle-Income Country with Universal Health Coverage

**DOI:** 10.1155/2020/7250250

**Published:** 2020-01-29

**Authors:** Kateir Contreras, Romar Baquero, Giancarlo Buitrago

**Affiliations:** ^1^Nephrology Unit, Department of Internal Medicine. School of Medicine, Universidad Nacional de Colombia, Bogotá D.C, Colombia; ^2^Hospital Universitario Nacional de Colombia, Bogotá D.C, Colombia; ^3^Clinical Research Seedbed. School of Medicine, Universidad Nacional de Colombia, Bogotá D.C, Colombia; ^4^Clinical Research Institute, Department of Surgery, Universidad Nacional de Colombia, Bogotá D.C, Colombia

## Abstract

Parathyroidectomy (PTX) is one of the most frequently performed surgeries in chronic kidney disease (CKD) patients. The objective of this study was to determine the intensive care unit (ICU) admission, mortality and hospital readmission rates within the 30-day postoperative period, and the total cost of the care episode and to determine possible prognostic factors in end-stage renal disease (ESRD) adult patients taken to PTX in the Colombian contributory health system. *Methods*. Retrospective cohort study of ESRD adult patients affiliated to the Colombian contributory health system, on dialysis for at least 3 months, undergoing PTX between January 1, 2012, and November 30, 2016. The clinical outcomes evaluated were rehospitalization at 30 days, hospital stay, and ICU requirement. The costs associated with the hospitalization event in which the PTX was performed from the perspective of the third payer were estimated. *Results*. The study included 478 patients. The mortality rate was 2.09 per 100 surgeries, the ICU admission rate was 32.64 per 100 surgeries, the 30-day hospital readmission percentage of the postoperative period was 16.74%, and the average length of hospital stay was 5.02 days. The median total costs of care for the entire procedure was USD $ 7,814.27 (p25-p75: 3,922.03–9,372.68), with significant regional differences. The geographical region was shown as a prognostic factor associated with clinical outcomes and the cost of care. *Conclusions*. There are large regional differences in readmission, ICU admission and mortality rates, and costs of dialysis ESRD patients undergoing PTX belonging to the Colombian contributory regime. The geographic region behaves as an independent predictor of clinical outcomes and costs.

## 1. Introduction

End-stage renal disease (ESRD) patients on chronic dialysis for more than 90 days have a high prevalence of alterations in bone mineral metabolism; secondary hyperparathyroidism (HPT) is the most frequent form, and is associated with adverse outcomes that include accelerated atherosclerosis, uraemic bone disease, refractory anemia, impaired quality of life, and increased mortality [1, 2]. Therapeutic interventions are based on calcium supplements, vitamin D analogues, calcimimetics, and parathyroidectomy (PTX) in cases refractory to medical treatment [2, 3]. The incidence of PTX is 8.09–14.2 cases per 1000 patient-years, and in those on renal replacement therapy (RRT) for more than 10 years, the incidence increases to 30 cases per 1000 patient-years [4]. This rate has remained essentially unchanged in recent years despite advances in medical therapy [5, 6]. However, it has been documented that PTX is associated with a higher risk of mortality, severe electrolyte alterations, higher hospital readmission rates, and requirement for intensive care unit (ICU) management, mainly in dialysis patients [7, 8].

Colombia is a medium-income country with universal health coverage (97%), and has two health system affiliation regimes. The subsidized regime is for people whose income is less than a minimum monthly salary, and the contributory regime for formal workers with higher incomes. In 2014, 47% of the total population was affiliated to the contributory regime [9]. Colombia is one of the few countries with a high universal health coverage rate in Latin America [10]. There are no differences by region or type of insurer in the health system coverage offered to ESRD patients.

ESRD is part of high-cost diseases, and payment is made in packages. PTX and the management of its complications are attended by event; however, clinical and paraclinical monitoring is performed in renal clinics. In addition, there are no reports on clinical outcomes and costs in this population in Latin America. The objective of this work is to describe clinical outcomes and costs associated with PTX in dialysis ESRD patients and to determine whether sociodemographic factors and comorbidities behave as a prognostic factor for these outcomes in Colombia, a medium-income country with universal health coverage.

## 2. Methods

### 2.1. Type of Study and Population

Retrospective cohort study based on administrative claims data using the basis for the study of the adequacy of the Capitation Payment Unit (CPU) of the Ministry of Health of Colombia. As mentioned in other studies, the CPU base corresponds to the information that the insurers of the Colombian Health System send to the Ministry of Health for the estimation of the premium that the system recognizes for each affiliate. This database is highly standardized, contains detailed information on all the services used by the contributory regime, and includes the identification of the type of service provided, an associated ICD-10 code, the date of service, the municipality, sex, age, identification of the insurer, and the service provider. Additionally, the death certificate database was used. The databases were anonymized, and the study was approved by the Ethics Committee of the School of Medicine of the Universidad Nacional de Colombia (evaluation report No. 020-333-18 of December 14, 2019).

### 2.2. Population

The study included all patients older than 18 years, with ESRD on dialysis for at least 3 months, undergoing PTX between January 1, 2012, and November 30, 2016, and who were affiliated to the contributory regime during all the years of the study.

### 2.3. Variables

The clinical outcomes evaluated were 30-day rehospitalization, hospital stay, and ICU requirement. Costs were estimated from the perspective of the third payer (*i.e.* Colombian health system), associated with the hospitalization event in which the PTX was performed. These costs included the value of the surgical procedure and all other costs derived from the care. The costs were deflated to 2016 US dollars. A distribution was made for five geographic regions: (1) Atlantic, which includes the departments of: Atlántico, Bolívar, Cesar, Córdoba, La Guajira, Magdalena, and Sucre; (2) Central: Antioquia, Caldas, Caquetá, Huila, Quindío, Risaralda, and Tolima; (3) Pacific: Valle del Cauca, Cauca, Chocó, and Nariño; (4) Eastern: Boyacá, Cundinamarca, Meta, Norte de Santander, Santander; Others: Arauca, Casanare, Putumayo, San Andrés, Amazonas, Guainía, Guaviare, Vaupés, and Vichada; and (5) Bogotá. In addition, the type of insurer to which the persons were affiliated was identified: public or private. Other possible prognostic factors were age, sex, service provider, and all the comorbidities reported in the Charlson index (CCI) at inclusion in the cohort [11].

### 2.4. Analysis

A description of the sociodemographic and clinical variables of the entire cohort was made. Mortality, ICU admission, and 30-day readmission rates for every 100 parathyroidectomies, as well as average stay and standard deviation (SD) of the total stay were estimated. The median and the 25th and 75th percentiles (p25-p75) of the total cost of care were also estimated. These outcome variables were presented by geographic regions, age categories, and type of insurer (public or private).

To determine the prognostic factors of the probability of ICU admission or 30-day hospital readmission, we performed a multivariate logistic regression model that included age, sex, geographic region, type of insurer, and CCI. The Colombian Pacific region was used as a comparison region, which is one with the worst indicators of economic development. In addition, a multivariate linear regression model was adjusted to determine the prognostic factors that are associated with the costs of care. All analyses were performed with Stata 15®.

## 3. Results

### 3.1. Descriptive

In the period of time described, 1815 PTXs were performed in the contributory health system, 478 of which were performed in ESRD patients who had been on chronic dialysis for more than 90 days, who were included in the study.  Table 1 presents the sociodemographic and clinical characteristics of these patients. The majority (49.79%) were between the ages of 40 and 59 years, 52.09% were women, and 60.46% of them had a CCI between 1 and 2. When categorizing by age, the CCI was higher in the subgroup of 50–60 years (59.65%); those under 49 years most frequently had 0–2 indexes (64.29%). The most frequent comorbidity was arterial hypertension (36.19%), followed by diabetes mellitus (16.74%); of these, the majority were older than 60 years. 4.81% of the patients had a history of renal transplantation (Table 2). 63.80% of the PTXs were carried out in the Bogotá and Central regions, and the public insurer predominated (52.30%).

The 30-day mortality rate for the entire cohort was 2.09 per 100 surgeries, the 30-day ICU admission rate was 32.64, the hospital readmission rate of the postoperative period was 16.74%, and the mean of the days spent in hospital was 5.02 (Table 3). The mortality rate decreased as age increased, with a range of 2.38 per 100 surgeries for the ≤40 year age category, and of 1.75 per 100 surgeries for the ≥60 year age category. The ICU admission rate was higher for the ≥60 year age category (35.09%) compared to patients aged ≤49 years (30.16%). Individuals with a CCI ≥3 had the highest mortality and rehospitalization rates. Finally, the highest mortality and hospital readmission rates were in the Central region; however, the highest ICU admission rate was found in the Pacific region. The public insurer showed worse outcomes compared to the private insurer.

### 3.2. Prognostic Factors

Tables 4 and 5 show the results of the multivariate logistic regression models that show the association between the geographic region and the type of insurer with the clinical outcomes adjusted for possible confounding variables. It is observed that, compared to the Pacific region, the *odds ratios* (ORs) of the other regions for 30-day ICU admission are less than 1 (Table 4). That is, the Pacific region is a poor prognosis factor for ICU admission. In contrast, regions other than the Pacific are poor prognosis factors for hospital readmission up to 30 days after discharge (Table 4).

In terms of 30-day postoperative ICU admissions, in the age and sex categories, ORs were not found to be determinant. And the ORs not adjusted with the logistic regression model did not show differences according to the CCI categories either. It was also found that being from any region other than the Pacific region was a protective factor. Likewise, it was found that patients with a public insurer have a greater risk of ICU admission in the postoperative period.

Regarding 30-day postoperative hospital readmissions, belonging to a public insurer increases the probability of ICU admission.

Table 5 presents the multilevel linear correlation coefficients for the association between the clinical and sociodemographic characteristics of the patients and the days spent in hospital. It was found that the geographical region did not behave as a risk factor for the hospital stay. On the contrary, being affiliated with a public insurer increases the average hospital stay.

The median total costs of care for the entire procedure was USD $ 7,814.27 (p25-p75: 3,922.03–9,372.68). An “U” (decreasing and increasing) tendency was observed in the 3 age groups upwardly, starting at USD $ 7,840.65 (p25-p75: 4,002.89–9,192.58) for patients aged ≤49 years, reaching the trough at USD $ 7,687.30 (p25-p75: 3,922.04–9,372.68) for patients over 49 years and under 60 years, and reaching a value of USD $ 8,050.20 (p25-p75: 3,793.20–9,993.62) in patients aged ≥60 years. With respect to geographical regions, the lowest median is in the eastern region (USD $ 5,733.51, p25-p75: 3,076.83–10,029.98), followed by other departments (USD $ 5,751.14, p25-p75: 5751.14–5,751.14), Atlantic (USD $ 6,817.58, p25-p75: 3,445.20–7,123.68), Bogotá (USD $ 7,349.4, p25-p75: 4,308.86–8,138.09), Central (USD $ 8,111.70, p25-p75: 3,587.30–10,029.98), and finally the Pacific region (USD) $ 10,307.87; p25-p75: 5,659.14–12,804.29).

Table 6 shows the multivariate linear correlation coefficients for the association between the clinical and sociodemographic characteristics of patients and the cost associated with health care. It was found that if the patient is part of the Atlantic (−2,945.47; −5,859.54; −31.41), Bogotá (−1,963.55; −3,675.82; −251.28), or Eastern (−3,847.27; −5,957.46; −1,737.08) regions, the procedure will cost less, compared with the Pacific region. Regarding the relationship with age, sex, and CCI, there was no correlation with difference in the cost of the procedure. The factor that did significantly increase the costs associated with health care was that of belonging to a public insurer (4.71, 2.06, 7.36).

## 4. Discussion

Using the national billing records of the contributory regime in a country with universal health coverage, this study was conducted in adult patients on RRT, and it was found that the 30-day postoperative readmission rate was 16.74%, the ICU admission rate was 32.64%, and the mortality rate was 2.09%. These results are similar to those found in the literature on large cohorts, and this is the first report on postoperative mortality associated with parathyroidectomy in Colombia and in a developing country.

PTX is one of the most frequent surgical procedures in the United States and the most frequent in ERSD patients [7]. It is a relatively safe procedure with minimally invasive surgery performed in reference centers [12], with mortality rates in the general population of 0.2% [13]. The most frequent indication is primary HPT (59.89%) by secretory adenoma, described in the general population, with some cases isolated in ERSD, the second indication of PTX is secondary HPT (21.9%), which is present in ERSD [7]. The PTX outcomes in dialysis patients are worse than in patients without CKD, and there is a higher probability of adverse events due to comorbidity: diabetes mellitus, hypertension, and heart failure [14]. Hospital readmissions and postparathyroidectomy mortality are 5 times more frequent than in the general population, with the subsequent impact on costs for health systems [7, 14]. Kuo et al. studied 898 dialysis patients and found 30-day readmission rates of 17.8%, mortality of 0.9%, and hospital stay of 4 days (interquartile range 2.6) [15]. In the cohort published by Kravietz A, 7171 PTXs were documented, and the 30-day postoperative readmission rate was 5.6% in cases of primary HPT and 19.4% in secondary HPT. The most frequent causes of readmission in the latter were hypocalcemia (22.88%) and hungry bone syndrome (14.38%) [7].

Ishani et al. used data from the US Renal Data System and identified 4435 PTXs performed on hemodialysis patients over 18 years of age. Mortality during PTX and in the first 30 days was 2%, the hospital readmission rate was 23.8%, and the ICU admission rate was 29.3% [16]. At 1-year follow-up, hospitalizations increased by 39%, as well as the days spent in hospital, and the emergency room visits for hypocalcaemia were 20 times higher than in the year prior to surgery [16]. Ferrandino et al. found 17.2% of readmissions, mainly due to hungry bone syndrome (40%), and found as risk factors for readmission, weight loss, and malnutrition at the time of surgery [14].

In the cohort that we present, no statistically significant differences were found in outcomes when performing the analysis by age and by CCI. At least five events are recommended for each of the independent variables that will be included in a multivariate logistic regression model [17]; due to the low number of deaths, the sample size of our study may be insufficient to find statistically significant estimators, when they actually exist (type II error).

Mortality seems to be higher in those under 50 years of age; however, when performing the analysis by subgroups, no differences were found in the CCI, therefore, we cannot attribute this mortality to greater comorbidity. Although it has been reported that young people have more severe HPT [18], since it is a study taken from administrative data, and we do not know the vintage dialysis, we cannot conclude this with our study.

In the multivariate model, the independent predictors of hospital readmission and ICU admission were sociodemographic: type of insurer and region of the country. Colombian health care system is an insurance system based on managed care competition. The Colombian Health System estimates an individual premium that is given annually to each insurer for the health services of its enrollees. Health insurers are responsible for administering the benefit plan. Insurers make contracts with health providers and offer a network to their enrollees. In different geographical regions or for some population segments (for example, with more unfavorable socioeconomic conditions), the only insurer available is the public insurer. Therefore, different social determinants of health with negative effects on health outcomes may be concentrated in the population that is covered by the public insurer. This phenomenon is also favored by strategies of private insurers that lead to the population's selection with the lowest health risks (i.e., with better health determinants, such as higher education level, higher income, residence in geographical locations with better sanitation services, etc.). This phenomenon is known as risk selection or “cream skimming.” Additionally, these determinants could be associated with worse quality or lack of continuity and opportunity in health care. We consider that this is the main explanation of why the public insurer and geographical region are independently associated with worse outcomes and higher costs [19–21]. The Pacific region showed great differences with respect to the rest of the country; although patients in that region had the lowest hospital readmission rates, the majority of them were admitted to ICU, which translates into higher costs. Given the study design, it is not possible to determine the cause of this difference and it should be the object of future research.

The median length of hospital stay in our study was 5.02 days, longer than the one reported in Kim SM's cohort, which was 3 days (25–75 percentile: 2–6 days) [22].

The median of the total costs of the PTX care episodes was USD $ 7814, which suggests that this intervention is less expensive in Colombia than in other countries [22], but with large regional differences and among insurers.

In Colombia, we adhere to international guidelines, mainly KDIGO [3]; in addition, each dialysis center has standardized management guidelines. Official sources report that the majority of patients are in 3 dialysis providers (77.3%) that meet the most high-quality standards [23].

In a country with universal health coverage, these regional differences are important, both in clinical and economic outcomes; these data have implications for the planning of care, the management of resources, and the implementation of public health policies. The data were obtained from the billing databases, which may cause limitations due to the omission of data. However, we know that these databases are updated frequently, cover the entire affiliated population, and reflect local medical practice. These data are recorded longitudinally from transactions that facilitate payments to health care providers [24, 25].

Although the contributory system does not represent the entire country, this study guarantees a high national representativeness of the population employed in the formal economic sector, which corresponds to 50% of the country's population. The other half of the population (subsidized system), which has more unfavorable socioeconomic conditions, is very likely to have worse results (higher readmission, mortality, and ICU admission rates).

Since these databases were not created specifically for research, they lack clinical and laboratory results data. We do not know the treatment that these patients received previously, the use of calcimimetics, or the dialysis window, data that could have an impact on the outcomes. We also do not know whether partial or complete PTX was performed. However, previous studies did not find differences in outcomes depending on the type of surgery [25]. The factors that may have influenced the sample size include the introduction of calcimimetics in Colombia from 2012, which may have reduced the number of parathyroidectomies.

The outcomes of our study and those reported in the literature show the need for the practice to be increasingly homogeneous, to optimize and monitor medical management and to use the therapeutic arsenal available before deciding to perform PTX. Currently, the accepted indications for PTX in secondary HPT are: persistent (more than 6 months) elevation of PTH >800 pg/mL despite pharmacological treatment with the maximum tolerated dose of vitamin D analogues and calcimimetics, or when PTH is between 600 and 800 pg/mL associated with persistent hypercalcemia or hyperphosphatemia (corrected calcium greater than 10.2 mg/dL, or phosphorus greater than 5.5 mg/dL) despite diet and treatment, high risk of calciphylaxis, and erythropoietin refractory anemia [26]. In view of the high incidence of hungry bone syndrome with potentially fatal outcomes in the perioperative period, the use of calcimimetics should be suspended, early calcium supplementation, vitamin D analogues should be initiated, and strict monitoring of calcium and phosphorus levels should be carried out during hospital stay and at discharge, and they should be monitored frequently in the renal unit to make the necessary adjustments, thereby seeking to reduce rehospitalization and mortality rates.

## 5. Conclusions

Patients with CKD on dialysis have poorer outcomes after PTX compared to the general population. It is associated with higher hospital readmission, ICU admission, and mortality rates. This is the first study conducted in Colombia and in a developing country. The predictors of independent risk were sociodemographic, with important differences by region and by the type of insurer in terms of outcomes and costs; these data are useful for the planning of care and for future research to identify the factors contributing to these disparities.

## Figures and Tables

**Table 1:** Patient demographic and preoperative characteristics.

Characteristic	*N* = 478
Age	
Median (p25; p75) (years)	49.44 (35.70; 63.17)
Groups (no. (%))	
≤49	126 (26.36)
50–59	238 (49.79)
≥60	114 (23.85)
Sex (no. (%))	
Male	229 (47.91)
Female	249 (52.09)
Comorbidity Charlson index (no. (%))	
0–2	289 (60.46)
≥3	189 (39.54)
Geographical region (no. (%))	
Atlantic	24 (5.02)
Bogotá	185 (38.70)
Central	120 (25.10)
Eastern	64 (13.39)
Pacific	84 (17.57)
Others departments	1 (0.21)
Insurer (no. (%))	
Public	250 (52.3)
Private	228 (47.7)

**Table 2:** Patient demographic and preoperative characteristics categorized by age.

Characteristic	Full sample	Age category		
		≤49 y	50–60 y	≥60 y
Comorbidity Charlson index (%)				
0–2	63.49	64.29	40.35	58.37
≥3	36.51	35.71	59.65	41.63
Comorbidity (%)				
Myocardial infarction	2.30	2.38	2.10	2.63
Congestive heart failure	5.23	7.14	3.78	6.14
Peripheral vascular disease	1.05	0.79	0.84	1.75
Cerebrovascular accident	3.97	3.17	5.04	2.63
Chronic obstructive pulmonary disease	7.95	3.17	6.72	15.79
Peripheral vascular disease	2.72	2.38	2.94	2.63
Liver disease	0.84	0.79	0.84	0.88
Diabetes mellitus	16.74	13.49	9.24	35.96
Solid tumor	14.44	9.52	14.71	19.30
Solid tumor metastatic	1.67	1.59	1.68	1.75
AIDS	2.09	0.79	2.94	1.75
Hypertension arterial	36.19	26.98	39.08	40.35
Kidney transplant	4.81	5.56	5.88	1.75
Observations	478	126	238	114

**Table 3:** Rate of 30-day postoperative mortality, ICU admissions, hospital readmission, cost, and days of hospital stay by age, comorbidities, and region.

Characteristic	Hospital readmissions/total (rate per 100 surgeries)	ICU admissions/total (%)	Hospital stay (days) (mean (SD)	Deaths/total (rate per 100 surgeries)	Cost USD (median (p25; p75))^a^
Age; groups					
≤49	21/126 (16.67)	38/126 (30.16)	5.19 (12)	3/126 (2.38)	7840.64 (4002.89; 9192.57)
50–59	40/238 (16.81)	78/238 (32.77)	4.71 (13.7)	5/238 (2.1)	7687.29 (3922.03; 9372.68)
≥60	19/114 (16.67)	40/114 (35.09)	5.51 (17.1)	2/114 (1.75)	8050.19 (3793.19; 9993.62)
Comorbidity Charlson index					
0–2	47/289 (16.26)	95/289 (32.87)	5.36 (16.5)	5/289 (1.73)	7898.29 (3690.63; 9386.52)
≥3	33/189 (17.46)	61/189 (32.28)	4.51 (9.4)	5/189 (2.65)	7685.78 (4330.29; 9309.39)
Geographical region					
Atlantic	1/24 (4.17)	8/24 (33.33)	4.58 (10.3)	0/24 (0)	6817.58 (3445.20; 7123.68)
Bogotá	33/185 (17.84)	45/185 (24.32)	4.37 (14)	4/185 (2.16)	7349.4 (4308.86; 8138.09)
Central	25/120 (20.83)	32/120 (26.67)	6.88 (19.7)	4/120 (3.33)	8111.70 (3587.30; 10029.98)
Eastern	12/64 (18.75)	12/64 (18.75)	2.56 (4.4)	1/64 (1.56)	5733.51 (3076.83; 7994.97)
Pacific	9/84 (10.71)	59/84 (70.84)	5.89 (9.9)	1/84 (1.19)	10307.87 (5659.14; 12804.29)
Others departments	0/1 (0)	0/1 (0)	1 (N/A)	0/1 (0)	5751.14 (5751.14; 5751.14)
Insurer					
Public	51/250 (20.4)	112/250 (44.8)	7.0 (18.1)	7/250 (2.8)	9177.28 (3922.03; 11423. 32)
Private	29/228 (12.7)	44/228 (19.3)	2.79 (7.1)	3/228 (1.32)	6319.73 (3245.58; 6985.41)
Total Colombia	80/478 (16.74)	156/478 (32.64)	5.02 (14.1)	10/478 (2.09)	7814 (3922.03; 9372.68)

^a^US DOLLARS 2016.

**Table 4:** Prognosis factors for 30-day postoperative ICU admission and hospital readmission.

Characteristic	30-day ICU admission^a^			30-day hospital readmission^a^		
	OR	(95%CI)	*p* value	OR	(95%CI)	*p* value
Age (years)	1.00	(0.98; 1.02)	0.79	1.00	(0.99; 1.02)	0.60
Sex						
Male	1.00			1.00		
Female	0.93	(0.61; 1.42)	0.74	1.27	(0.77; 2.09)	0.34
Comorbidity Charlson index						
0–2	1.00			1.00		
≥3	0.94	(0.60; 1.46)	0.79	1.03	(0.62; 1.72)	0.90
Geographical region						
Pacific	1.00			1.00		
Atlantic	0.25	(0.09; 0.67)	0.01	0.41	(0.05; 3.45)	0.41
Bogotá	0.18	(0.10; 0.33)	0.00	2.33	(1.03; 5.24)	0.04
Central	0.20	(0.10; 0.37)	0.00	2.81	(1.21; 6.52)	0.02
Eastern	0.18	(0.05; 0.26)	0.00	2.40	(1.21; 6.52)	0.07
Others departments	NA		NA			
Insurer						
Private	1.00			1.00		
Public	2.52	(1.62; 3.93)	0.00	2.06	(1.23; 3.46)	0.01

NA: not applicable (no ICU admissions or no readmission). ^a^Multivariate logistic regression analysis.

**Table 5:** Prognostic factors for hospital stay.

Characteristic	Multivariate adjustment^a^		
	Coefficient^b^	95%CI	*p* value
Age (years)	−0.03	(0.012; 0.06)	0.53
Sex			
Male	Reference		
Female	−1.97	(−4.52; 0.57)	0.13
Comorbidity Charlson index			
0–2	Reference		
≥3	−1.13	(−3.79; 1.52)	0.40
Geographical region			
Pacific	Reference		
Atlantic	−0.28	(−6.70; 6.15)	0.93
Bogotá	0.36	(−3.41; 4.14)	0.85
Central	2.50	(−1.53; 6.52)	0.22
Eastern	−2.11	(−6.76; 2.54)	0.37
Others departments	−2.71	(−30.57; 25.16)	0.85
Insurer			
Private	Reference		
Public	4.71	2.06; 7.36	0.00

^a^Multivariate linear regression model. ^b^Days. Costs associated with health care.

**Table 6:** Prognostic factors for health care costs related to patients with CKD taken to parathyroidectomy.

Characteristic	Multivariate adjustment^a^		
	Coefficient^b^	95%CI	*p* value
Age (years)	−6.91	(−49.68; 35.85)	0.75
Sex			
Male	Reference		
Female	−364.71	(−1519.09; 789.67)	−0.62
Comorbidity Charlson index			
0–2	Reference		
≥3	−387.48	(−1591.99; 817.02)	0.53
Geographical region			
Pacific	Reference		
Atlantic	−2,945.47	(−5,859.54; −31.41)	0.05
Bogotá	−1,963.55	(−3,675.82; −251.28)	0.03
Central	−1,338.74	(−3,166.30; 488.82)	0.15
Eastern	−3,847.27	(−5,957.46; −1,737.08)	0.00
Others departments	−2,871.16	(−15,513.74; 9,771.43)	0.66
Insurer			
Private	Reference		
Public	4.71	2.06; 7.36	0.00

Multivariate linear regression model. ^b^2016 US dollars.

## Data Availability

The UPC data used to support the findings of this study were supplied by Colombian Minister of Health and Social Protection under agreement to Universidad Nacional de Colombia and so cannot be made freely available. Requests for access to these data should be made to Office of Information and Communication Technology of Colombian Ministry of Health and Social Protection.

## References

[1] Pitt S. C., Sippel R. S., Chen H. (2009). Secondary and tertiary hyperparathyroidism, state of the art surgical management. *Surgical Clinics of North America*.

[2] Chen J.-B., Chou F.-F., Yang C.-H., Hua M.-S. (2017). Association between clinical variables and mortality after parathyroidectomy in maintenance hemodialysis patients. *The American Journal of Surgery*.

[3] Ketteler M., Block G. A., Evenepoel P. (2017). Executive summary of the 2017 KDIGO chronic kidney disease-mineral and bone disorder (CKD-MBD) guideline update: what’s changed and why it matters. *Kidney International*.

[4] Malberti F., Marcelli D., Conte F., Limido A., Spotti D., Locatelli F. (2001). Parathyroidectomy in patients on renal replacement therapy: an epidemiologic study. *Journal of the American Society of Nephrology*.

[5] Foley R. N., Li S., Liu J., Gilbertson D. T., Chen S.-C., Collins A. J. (2004). The fall and rise of parathyroidectomy in U.S. Hemodialysis patients, 1992 to 2002. *Journal of the American Society of Nephrology*.

[6] Li S., Chen Y.-W., Peng Y., Foley R. N., St. Peter W. L. (2011). Trends in parathyroidectomy rates in US hemodialysis patients from 1992 to 2007. *American Journal of Kidney Diseases*.

[7] Kravietz A. M., Buicko J. L., Parreco J. P., Lopez M. A., Kozol R. A. (2018). Thirty-day readmissions following parathyroidectomy: evidence from the national readmissions database, 2013-2014. *American Journal of Otolaryngology*.

[8] Anderson J. E., Olson J. L., Campbell M. J. (2016). Parathyroidectomy in dialysis patients: what is the risk?. *World Journal of Endocrine Surgery*.

[9] OECD (2015). *OECD Reviews of Health Systems: Colombia 2016*.

[10] Atun R., De Andrade L. O. M., Almeida G. (2015). Health-system reform and universal health coverage in Latin America. *The Lancet*.

[11] Brusselaers N., Lagergren J. (2017). The Charlson comorbidity index in registry-based research. *Methods of Information in Medicine*.

[12] Morris L. F., Lee S., Warneke C. L. (2014). Fewer adverse events after reoperative parathyroidectomy associated with initial minimally invasive parathyroidectomy. *The American Journal of Surgery*.

[13] Gupta P. K., Smith R. B., Gupta H., Forse R. A., Fang X., Lydiatt W. M. (2012). Outcomes after thyroidectomy and parathyroidectomy. *Head & Neck*.

[14] Ferrandino R., Roof S., Ma Y. (2017). Unplanned 30-day readmissions after parathyroidectomy in patients with chronic kidney disease: a nationwide analysis. *Otolaryngology-Head and Neck Surgery*.

[15] Kuo L. E., Wachtel H., Karakousis G., Fraker D., Kelz R. (2014). Parathyroidectomy in dialysis patients. *Journal of Surgical Research*.

[16] Ishani A., Liu J., Wetmore J. B. (2015). Clinical outcomes after parathyroidectomy in a nationwide cohort of patients on hemodialysis. *Clinical Journal of the American Society of Nephrology*.

[17] Peduzzi P., Concato J., Kemper E., Holford T. R., Feinstein A. R. (1996). A simulation study of the number of events per variable in logistic regression analysis. *Journal of Clinical Epidemiology*.

[18] Wetmore J. B., Liu J., Dluzniewski P. J., Ishani A., Block G. A., Collins A. J. (2016). Geographic variation of parathyroidectomy in patients receiving hemodialysis: a retrospective cohort analysis. *BMC Surgery*.

[19] Barros P. P. (2003). Cream-skimming, incentives for efficiency and payment system. *Journal of Health Economics*.

[20] Van De Ven W. P. M. M., Beck K., Buchner F. (2003). Risk adjustment and risk selection on the sickness fund insurance market in five European countries. *Health Policy*.

[21] Buitrago G., Junca E., Eslava-Schmalbach J., Caycedo R., Pinillos P., Leal L. C. (2019). Clinical outcomes and healthcare costs associated with laparoscopic appendectomy in a middle-income country with universal health coverage. *World Journal of Surgery*.

[22] Kim S. M., Long J., Montez-Rath M. E., Leonard M. B., Norton J. A., Chertow G. M. (2016). Rates and outcomes of parathyroidectomy for secondary hyperparathyroidism in the United States. *Clinical Journal of the American Society of Nephrology*.

[23] Fondo Colombiano para las enfermedades de alto costo -Cuenta de alto costo (2017). *Situación de la Enfermedad Renal Crónica, la Hipertensión Arterial y la Diabetes Mellitus en Colombia*.

[24] Lin K., Schneeweiss S. (2016). Considerations for the analysis of longitudinal electronic health records linked to claims data to study the effectiveness and safety of drugs. *Clinical Pharmacology & Therapeutics*.

[25] Rassen J. A., Bartels D. B., Schneeweiss S., Patrick A. R., Murk W. (2019). Measuring prevalence and incidence of chronic conditions in claims and electronic health record databases. *Clinical Epidemiology*.

[26] Lau W. L., Obi Y., Kalantar-Zadeh K. (2018). Parathyroidectomy in the management of secondary hyperparathyroidism. *Clinical Journal of the American Society of Nephrology*.

